# The Atypical Face of Neuroleptic Malignant Syndrome: A Case Report of Ileus and Absent Rigidity

**DOI:** 10.7759/cureus.63784

**Published:** 2024-07-03

**Authors:** Tarun Kutumbaka, Sivaprakash Varadan, R. B. Sudagar Singh

**Affiliations:** 1 General Medicine, Sri Ramachandra Institute of Higher Education and Research, Chennai, IND; 2 Internal Medicine, Sri Ramachandra Institute of Higher Education and Research, Chennai, IND

**Keywords:** drug-induced hyperthermia, rhabdomyolysis, paralytic ileus, antipsychotic medication, neuroleptic malignant syndrome (nms)

## Abstract

Neuroleptic malignant syndrome (NMS) is a rare life-threatening condition associated with the use of antipsychotic medications. This case report describes a male patient in his early 30s who presented with fever, breathlessness, and lower limb weakness, ultimately diagnosed with NMS despite the absence of muscular rigidity. On examination, he was febrile, tachycardic, and tachypneic with an oxygen saturation of 88% and elevated blood pressure. On auscultation diffuse crepitations in both lungs were revealed. Neurological assessment indicated motor strength of 3/5 in both lower limbs, without rigidity, sensory loss, or cerebellar signs. It was noted that he was on irregular atypical antipsychotic medication for the past one year. Laboratory investigations revealed leukocytosis, elevated transaminases, dyselectrolytemia, elevated creatine phosphokinase (CPK), and serum creatinine. NMS was not initially considered due to the lack of muscular rigidity. However, the patient later developed autonomic dysregulation manifestations, such as paralytic ileus. Once organic causes were excluded, NMS was diagnosed. Supportive therapy included 23 cycles of hemodialysis and colonic decompression for pseudo-obstruction. He was treated with intravenous fluids and dopamine receptor agonist medications. NMS usually presents with fever, muscular rigidity, altered mental status, and autonomic instability; yet, the absence of muscular rigidity in this patient is a distinctive and unusual feature.

## Introduction

Neuroleptic malignant syndrome (NMS) is a rare potentially fatal condition triggered by antipsychotic drugs or withdrawal of dopamine receptor agonists. The incidence among antipsychotic users ranges from 0.02% to 3% [1]. NMS is classically defined by the tetrad of fever, altered mental status, rigidity, and autonomic dysfunction. However, it can present atypically, complicating diagnosis and treatment. NMS can develop even after just one dose or following prolonged use of the same antipsychotic agent, as it is not dependent on dosage. Although higher doses may increase the risk, NMS can still occur at low doses if certain risk factors are present [2]. Some atypical cases may present with a milder form of muscular rigidity or even with its absence [3-4] and colonic pseudo-obstruction [5]. The proposed mechanism involves dopamine dysregulation [6] primarily affecting the hypothalamus and basal ganglia, resulting in symptoms such as hyperthermia, autonomic dysfunction, and severe muscular rigidity or tremors. However, there are many other clinical conditions that can have similar clinical presentations. Therefore, NMS is primarily diagnosed through the exclusion of other possible conditions. Nierenberg and colleagues’ criteria are used for diagnosis [3]. The most common complications were rhabdomyolysis and others are acute kidney injury (AKI), acute respiratory failure, and sepsis [7]. Treatment includes stopping the causative agent and supportive care in the ICU setting with aggressive hydration therapy while maintaining a euvolemic state. Bromocriptine, amantadine, and dantrolene are commonly used agents [8]. Complicated AKI requires hemodialysis [9]. In some cases of severe NMS and where underlying psychotic depression or catatonia is present, electroconvulsive therapy (ECT) may be preferred [10]. The absence of muscle rigidity in this instance of NMS, associated with atypical antipsychotics, is a significant aspect highlighted by this case.

## Case presentation

A 36-year-old male with a one-year history of delusional disorder was brought to the emergency room by his uncle after being found lying on the floor early in the morning. His medical history revealed that he had been taking oral medications: Clozapine 50 mg/day, Risperidone 3 mg/day, and Amitriptyline 10 mg/day. However, he stopped these medications five days before admission due to personal stress. He resumed his medications the night before presenting to the emergency room. There was no previous history of similar complaints or adverse reactions to medications.

On arrival, he was irritable. His vitals were recorded as follows: temperature 104.8°F, blood pressure 150/90 mmHg, pulse rate 132 beats/min, and oxygen saturation 88% in room air. Clinical findings revealed diffuse crepitations in both lungs on auscultation. Central nervous system examination showed normal pupillary reaction, motor strength of 3/5 in both lower limbs, normal tone, reflexes, sensory system, and intact cerebellar signs. High-colored urine and oliguria were noted, which later progressed to anuria. He exhibited persistent tachycardia, sweating, and unstable blood pressure (systolic: 100-170 mmHg; diastolic: 60-100 mmHg). Initial laboratory and other investigations are mentioned in Table 1 and Table 2, respectively.

**Table 1:** Laboratory investigations Hb: hemoglobin; WBC: white blood cells; PCV: packed cell volume; BUN: blood urea nitrogen; TSH: thyroid stimulating hormone; SGOT: serum glutamic oxaloacetic transaminase; SGPT: serum glutamate pyruvate transaminase

CBC	Hb	WBC	PCV	Platelets		
	15.8 g/dL (13-15 g/dL)	25,500 cells/cu.mm (4000-10000 cells/cu.mm)	45.3 L/L (30-50 L/L)	2.27 lakhs/cu.mm (1.5 to 4.5 lakhs/cu.mm)		
Renal function test	BUN	Creatinine	Sodium	Potassium	Chloride	Bicarbonate
	28 mg/dL (7.9-20 mg/dL)	2.6 mg/dL (0.8-1.3 mg/dL)	110 mol/L (135-145 m.mol/L)	4.6 mol/L(3.5-4.5 m.mol/L)	78 mol/L (96-109 m.mol/L)	17 mol/L (21-31 m.mol/L)
	Uric acid	Phosphorus	Calcium	Free T3	Free T4	TSH
	121 mg/dL (2.6-7.2 mg/dL)	10.4 mg/dL (2.5-4.9 mg/dL)	6.6 mg/dL (8.5-10.1 mg/dL)	1.08 pg/mL (2-4.4 pg/mL)	1.18 ng/dL (0.93-1.7 ng/dL)	2.5 micro IU/mL (0.27-4.2 micro IU/mL)
Liver function test	SGOT	SGPT	Total bilirubin	Total protein	Alkaline phosphatase	
	2666 U/L (<50 U/L)	1060 U/L(<50 U/L)	1.20 mg/dL (0.3-1.2 mg/dL)	6.8 g/dL (6.6-8.3 g/dL)	73 IU/L (32-122 IU/L)	
Urine myoglobin	Positive					

**Table 2:** Other investigations

Other investigations
The respiratory panel revealed negative results for SARS-CoV-19, H1N1, influenza A and B
The results of the tropical viral fever panel for malaria, dengue, scrub, leptospirosis, and other illnesses were negative
HIV, hepatitis B, and hepatitis C viral markers came out negative
Urine culture and 3 sets of blood cultures showed no organism growth
Cerebrospinal fluid (CSF) analysis was normal

Radiological data are mentioned in Table 3.

**Table 3:** Radiological data MRI: magnetic resonance imaging; CECT: contrast-enhanced computed tomography

Test	Findings
Ultrasound abdomen	No significant abnormalities. No evidence of hepatosplenomegaly
MRI brain	No significant abnormalities
CECT abdomen	A dilated transverse colon measuring 10.5 cm was seen, and the collapsed appearance of the intestine distal to the descending colon suggested colonic pseudo-obstruction

**Figure 1:**
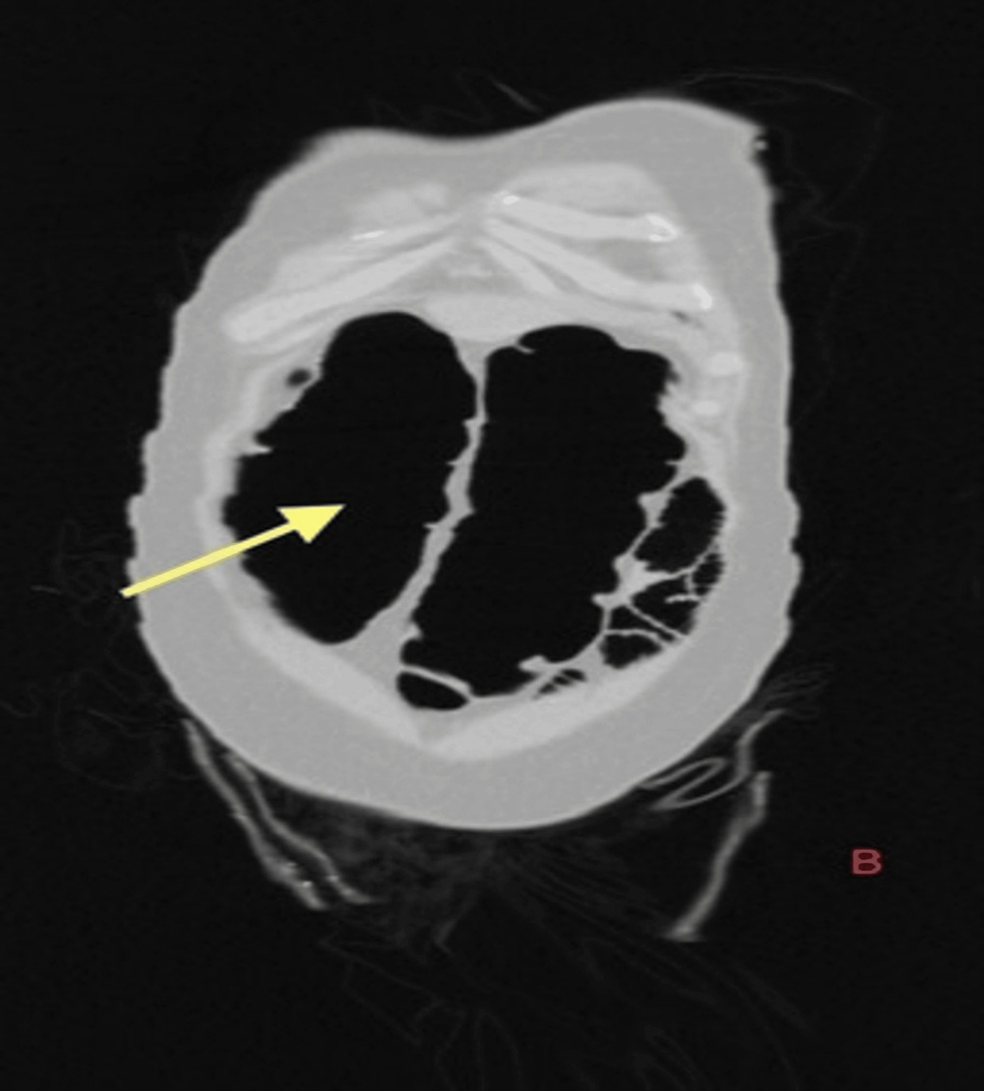
CECT abdomen (coronal plane) showing dilated transverse colon (yellow arrow) of 10.5 cm with normal caliber cecum, ascending colon, and proximal descending colon. The bowel from the distal descending colon appears collapsed CECT: contrast-enhanced computed tomography

During the first nine days of admission, the patient was constipated despite the administration of laxatives and enemas. Gradually, he developed abdominal distension. Clinical examination revealed overall abdominal tenderness. Radiological investigation (Figure 1) identified colon pseudo-obstruction.

Antipsychotic medications were discontinued, and antipyretic measures were implemented. Serial monitoring of serum creatinine and creatine phosphokinase (CPK) (Table 4) was done.

**Table 4:** Serial serum creatinine and creatine phosphokinase monitoring during the course of admission

Day of admission	serum creatinine in mg/dL (normal range: 0.8-1.3 mg/dL)	serum creatine phosphokinase in U/L (normal range: 20-200 U/L)
1	2.6	1,12,536
2	4	1,16,265
4	7.4	79,270
11	9.8	1888
13	10.2	737
16	7.9	80
20	10.9	
25	6	
35	5.5	
45	2.5	
49	1.7	
60	1.2	

However, persistent anuria and elevated serum creatinine, necessitate continued hemodialysis. After 14 cycles of hemodialysis on day 22 of illness, urine output increased to 50 ml/day and gradually improved with subsequent sessions. A total of 23 hemodialysis sessions were given for AKI during the illness. Regarding colonic pseudo-obstruction, attempts with a colonoscope failed beyond the sigmoid colon, necessitating decompression with a flatus tube to prevent perforation. Bladder and bowel dysfunction were successfully resolved. During the hospital stay, the patient received a daily dose of 20 mg of Bromocriptine tablets in divided doses for three weeks until his clinical condition improved, later tapered to 15 mg per day in divided doses for two weeks. At discharge, on day 49 of illness, serum creatinine level was 1.7. The patient was prescribed oral medications: Bromocriptine 2.5 mg every six hours (10 mg per day) and Diazepam 5 mg at bedtime, which facilitated clinical improvement. On follow-up, his creatinine was 1.2 mg/dL (normalized). Oral medications Bromocriptine and Diazepam were stopped.

## Discussion

The differential diagnosis considered included rhabdomyolysis myoglobinuria secondary to infectious etiology, NMS, heat stroke, serotonin syndrome, and malignant hyperthermia. Neuroimaging, cerebrospinal fluid analysis, viral markers, respiratory and tropical viral fever panels, along with blood and urine culture sensitivity, were negative, ruling out infectious etiology. In heat stroke, patients typically present with orthostatic hypotension without autonomic instability features. In this case, the patient's blood pressure was elevated, and he exhibited dysautonomia, including features like Ogilvie's syndrome, making heat stroke less likely. Serotonin syndrome was excluded due to the absence of diarrhea, raised CPK levels, and the lack of hyperreflexia or clonus. Differentiation from malignant hyperthermia was based on the absence of exposure to triggering factors such as depolarizing neuromuscular junction blockers and rigidity [11].

The specific criteria used for diagnosing typical NMS is the Diagnostic and Statistical Manual of Mental Disorders - Fifth Edition, Text Revision (DSM-5-TR). Nierenberg and colleagues’ criteria [3] can be used for diagnosing atypical NMS. However, in our case, it satisfied Nierenberg and colleague’s criteria with the presence of essential criteria (received a neuroleptic drug) and four major criteria (hyperthermia without other cause, elevated serum CPK, altered consciousness, autonomic instability such as tachycardia and labile blood pressure). Flexible criteria such as Levenson’s or Adityanjee and Aderibigbe are more adaptable compared to DSM-5-TR [11] in diagnosing atypical NMS. According to Adityanjee and Aderibigbe's criteria, our case was classified as type II NMS. Given the similarity of symptoms between NMS and other systemic or neurological diseases, the differential diagnoses must be thoroughly ruled out prior to confirming NMS, which enhances diagnostic accuracy. As described in our patient scenario, it is very difficult to suspect or diagnose the neuroleptic malignant syndrome with no rigidity despite high-grade temperature unless we know little literature on these presentations. There are few studies reported to have 10-40% of NMS cases caused by atypical antipsychotics presenting either fever or rigidity, otherwise milder forms of both [12]. So, in the absence of such a major characteristic, it leads to delayed diagnosis and treatment.

The patient developed critical dysautonomic features like paralytic ileus shortly after presentation, possibly due to clozapine’s anticholinergic effects or as a sign of NMS. Pathophysiologically reduced dopaminergic activity in the hypothalamus causes autonomic dysregulation [13]. There were some case reports by Tanaka et al. [14] and Lo et al. [15] have noted paralytic ileus preceding or following other NMS symptoms suggesting it should be considered in differential diagnoses of acute abdomen, with surgeons aware of neuroleptic-treated patients presenting with ileus. Acute tubular necrosis caused by myoglobinuria requires renal supportive therapy such as hemodialysis. Since the rhabdomyolysis in our case is so severe, the patient was continued on intermittent hemodialysis during the clinical course. In this patient, even though the fever subsided and CK levels normalized, the clinical picture was unstable. So in need of accelerated improvement, oral bromocriptine was added and titrated accordingly.

## Conclusions

In conclusion, this case highlights the importance of considering NMS in patients presenting with fever, autonomic instability, and elevated CPK, even in the absence of muscular rigidity. Early recognition and comprehensive supportive care, including hemodialysis and management of dysautonomic complications, are crucial for recovery. This case underscores the need for heightened clinical awareness of atypical presentations of NMS associated with atypical antipsychotic medications.

## References

[1] Velamoor VR (1998). Neuroleptic malignant syndrome. Recognition, prevention and management. Drug Saf.

[2] Eroğlu EÖ, Yildiz Mİ, Yazici MK (2021). Atypical neuroleptic malignant syndrome induced by low dose quetiapine in a patient treated with donepezil. Noro Psikiyatr Ars.

[3] Özdemir İ, Kuru E, Safak Y, Tulacı RG (2018). A neuroleptic malignant syndrome without rigidity. Psychiatry Investig.

[4] Vellekkatt F, Kuppili PP, Bharadwaj B, Menon V (2019). Atypical neuroleptic malignant syndrome - a case report. Asian J Psychiatr.

[5] Chime C, Alemam A, Kumar K, Dhallu M (2018). Acute abdomen with ileus: a heralding presentation of neuroleptic malignant syndrome. Case Rep Gastroenterol.

[6] Spivak B, Gonen N, Mester R, Averbuch E, Adlersberg S, Weizman A (1996). Neuroleptic malignant syndrome associated with abrupt withdrawal of anticholinergic agents. Int Clin Psychopharmacol.

[7] Modi S, Dharaiya D, Schultz L, Varelas P (2016). Neuroleptic malignant syndrome: complications, outcomes, and mortality. Neurocrit Care.

[8] Pileggi DJ, Cook AM (2016). Neuroleptic malignant syndrome. Ann Pharmacother.

[9] Chang CK, Payus AO, Noh MM, Lansing MG, Sumpat D, Lu SJ, Yew BT (2022). Neuroleptic malignant syndrome secondary to olanzapine, a presentation with severe acute kidney injury requiring hemodialysis: a case report. J Med Case Rep.

[10] Trollor JN, Sachdev PS (1999). Electroconvulsive treatment of neuroleptic malignant syndrome: a review and report of cases. Aust N Z J Psychiatry.

[11] Szota AM, Radajewska I, Araszkiewicz AS (2022). Atypical neuroleptic malignant syndrome: case reports and diagnostic challenges. J Psychoactive Drugs.

[12] Ananth J, Parameswaran S, Gunatilake S, Burgoyne K, Sidhom T (2004). Neuroleptic malignant syndrome and atypical antipsychotic drugs. J Clin Psychiatry.

[13] Pelonero AL, Levenson JL, Pandurangi AK (1998). Neuroleptic malignant syndrome: a review. Psychiatr Serv.

[14] Tanaka O, Otani K, Kondo T, Kaneko S, Fukushima Y (1993). Paralytic ileus as a prodromal symptom of the neuroleptic malignant syndrome. Hum Psychopharm Clin.

[15] Lo TC, Unwin MR, Dymock IW (1989). Neuroleptic malignant syndrome: another medical cause of acute abdomen. Postgrad Med J.

